# Alterations in Rat Accumbens Dopamine, Endocannabinoids and GABA Content During WIN55,212-2 Treatment: The Role of Ghrelin

**DOI:** 10.3390/ijms22010210

**Published:** 2020-12-28

**Authors:** Chrysostomos Charalambous, Marek Lapka, Tereza Havlickova, Kamila Syslova, Magdalena Sustkova-Fiserova

**Affiliations:** 1 Department of Addictology, First Faculty of Medicine, Charles University, Apolinarska 4, 128 00 Prague 2, Czech Republic; chrysostomos.charalambous@lf1.cuni.cz; 2 Department of Pharmacology, Third Faculty of Medicine, Charles University, Ruska 87, 100 34 Prague 10, Czech Republic; marek.lapka@centrum.cz (M.L.); terez.hav@gmail.com (T.H.); 3 Laboratory of Medicinal Diagnostics, Department of Organic Technology, University of Chemistry and Technology Prague, Technicka 5, 166 28 Prague 6, Czech Republic; kamila.syslova@vscht.cz

**Keywords:** synthetic cannabinoid WIN55,212-2, endocannabinoids, anandamide/AEA, 2-arachidonoylglycerol/2-AG, dopamine, dopamine metabolism, GABA, ghrelin/GHS-R1A, addiction, nucleus accumbens shell microdialysis

## Abstract

The endocannabinoid/CB1R system as well as the central ghrelin signalling with its growth hormone secretagogoue receptors (GHS-R1A) are importantly involved in food intake and reward/reinforcement processing and show distinct overlaps in distribution within the relevant brain regions including the hypothalamus (food intake), the ventral tegmental area (VTA) and the nucleus accumbens (NAC) (reward/reinforcement). The significant mutual interaction between these systems in food intake has been documented; however, the possible role of ghrelin/GHS-R1A in the cannabinoid reinforcement effects and addiction remain unclear. Therefore, the principal aim of the present study was to investigate whether pretreatment with GHS-R1A antagonist/JMV2959 could reduce the CB1R agonist/WIN55,212-2–induced dopamine efflux in the nucleus accumbens shell (NACSh), which is considered a crucial trigger impulse of the addiction process. The synthetic aminoalklylindol cannabinoid WIN55,212-2 administration into the posterior VTA induced significant accumbens dopamine release, which was significantly reduced by the 3 mg/kg i.p. JMV2959 pretreatment. Simultaneously, the cannabinoid-increased accumbens dopamine metabolic turnover was significantly augmented by the JMV2959 pretreament. The intracerebral WIN55,212-2 administration also increased the endocannabinoid arachidonoylethanolamide/anandamide and the 2-arachidonoylglycerol/2-AG extracellular levels in the NACSh, which was moderately but significantly attenuated by the JMV2959 pretreatment. Moreover, the cannabinoid-induced decrease in accumbens γ-aminobutyric acid/gamma-aminobutyric acid levels was reversed by the JMV2959 pretreatment. The behavioural study in the LABORAS cage showed that 3 mg/kg JMV2959 pretreatment also significantly reduced the systemic WIN55,212-2-induced behavioural stimulation. Our results demonstrate that the ghrelin/GHS-R1A system significantly participates in the rewarding/reinforcing effects of the cannabinoid/CB1 agonist that are involved in cannabinoid addiction processing.

## 1. Introduction

Cannabinoids enhance the subjective sense of well-being by stimulating the mesolimbic/mesocortical endocannabinoid system, which influences the motivation for natural rewards (such as palatable food, social interaction and sexual activity) and modulates the rewarding effects of addictive drugs. Prolonged activation of the endocannabinoid system by cannabis/cannabinoids can elicit neuroadaptations which may lead to addiction and other adverse consequences [1,2,3,4]. The endocannabinoid system consists of central and peripheral effects, several binding sites and numerous endogenous ligands [5,6,7]. The most studied endocannabinoids, arachidonoylethanolamide/anandamide (AEA) and 2-arachidonoylglycerol (2-AG), are synthetised on demand from the cell phospholipids and through cannabinoid CB1 receptors, retrogradely regulating synaptic neurotransmissons (e.g., glutamate, γ-aminobutyric acid/gamma-aminobutyric acid (GABA)) within the brain’s reward circuitry, controlling both excitatory (glutamate) and inhibitory (GABA) inputs [2,3,8]. The endocannabinoid system acts as an important mediator of synaptic plasticity in the mesocorticolimbic/corticostriatal pathways which are involved in motivated behaviour control [1,3,9].

The CB1Rs are the most abundant G protein-coupled receptors expressed in the adult brain with particular dense expression in regions involved in reward, addiction and cognitive functions including the ventral tegmental area (VTA), nucleus accumbens (NACs) and hippocampus [3,10,11]. The CB2Rs have also been recently found in the midbrain dopamine neuron regions. It seems that the CB1Rs’ activation produces reinforcing effects, whereas the CB2Rs’ produces aversive ones [1,12]. Cannabinoids including the main psychoactive constituent of cannabis, tetrahydrocannabinol (THC) as well as the synthetic aminoalkylindol cannabinoid WIN55,212-2, show typical biphasic/dual effects, with reinforcing and hyperactivity-stimulating low doses but aversive and hypoactivity-inducing high doses. Tetrahydrocannabinol is considered a partial CB1R/CB2R agonist and WIN55,212-2 a full CB1R/CB2R agonist [2,12,13,14].

All addictive drugs significantly activate dopaminergic transmission in the nucleus accumbens shell (NACSh), which is considered an important initial impulse of the addiction processes, linked with reward, reinforcement and disruption of salience attribution [15,16,17,18]. Tetrahydrocannabinol, WIN55,212-2 as well as AEA and 2-AG, through mesolimbic CB1Rs, increase dopamine concentration in the NACSh which is followed by reinforcement, conditioning and salience alteration processing [2,3,4,19,20,21,22,23]. The CB1Rs are located mainly on presynaptic GABAergic and glutamatergic axons within the VTA and the NAC (less on dendrites and soma), and their activation restricts neurotransmitter release and modulates neural firing [1,3]. These modulatory mechanisms are used by endocannabinoids (AEA, 2-AG) that are synthesized/released “on demand” by postsynaptic neurons (e.g., by dopaminergic neurons in the VTA and GABAergic medium spiny neurons (MSNs) in the NACSh) during heightened neural activity and, in a retrograde manner, inhibit presynaptic neurotransmitter release, thus causing depolarisation-induced suppression of inhibition (DSI, e.g., reduced GABA release) or excitation (DSE, e.g., reduced glutamate release). The CB1Rs activation by (endo)cannabinoids can also initiate long-term synaptic plasticity including long-term depression (LTD) and potentiation (LTP) [1,3]. Within the VTA, the dopamine neurons are muted by GABA (through GABA-A or GABA-B receptors); thus, (endo)cannabinoid/2-AG -induced DSI causes dopamine neuron disinhibition that provokes dopamine release in the NACSh. In addition to GABA, CB1R activation directly inhibits the release of glutamate and acetylcholine, which further produces widespread effects on neural signalling across many neurotransmitter systems. Furthermore, suggested cooperation of (endo)cannabinoids with other neurotransmitter systems are based on the CB1R oligomerisation and/or dimerization with dopaminergic D2R, opioid µ and δ OR, serotonin 2A receptor/5HT2AR, etc. [2,24]. The cannabinoid-induced accumbens dopamine efflux was reversed by systemic and intra-VTA opioid antagonist naloxone, possibly through modulation of VTA GABA neurons [20,25] and by drugs that decrease corticostriatal glutamatergic neurotransmission and in extend the glutamate-dependent dopamine release in the striatum, such as adenosine A2A antagonists or α7 nicotinic acetylcholine receptor antagonists [26].

Recently, significant mutual interactions between the endogenous cannabinoid system and the ghrelin system were documented in the regulation of food intake on a base homeostatic level as well as hedonic principles [27,28,29,30]. The G protein-coupled GHS-R1A is widely expressed throughout both the peripheral organs and the brain. It is notable for its high level of constitutive activity; thus, in the absence of ligand, it has its physiological roles [31,32]. Acylated ghrelin, when bound to the GHS-R1A, plays an important role in many fundamental functions and has complex interrelationships with a multitude of other systems [33,34]. Growth hormone secretagogoue receptors’ oligomerisations and dimerizations with a wide array of other G protein-coupled receptors have been found, including dopamine D1R and D2R, serotonin 2C receptor (5-HT-2CR), etc., and the existence of a cannabinoid CB1R-type heterodimer has been suggested [30,34]. Midbrain GHS-R1As are co-localized with dopaminergic and cholinergic receptors [35,36]; they functionally interact in amplification of the dopaminergic signalling in the VTA neurons and stimulate accumbens dopamine release [37,38]. The ability of GHS-R1A to heterodimerize with D1R/D2R in the VTA and the constitutive activity of the GHS-R1A possibly alter the sensitivity of the mesolimbic dopamine system [31,39]. Ghrelin and its GHS-R1A receptors and also cannabinoid CB1Rs are distributed within overlapping brain regions which participate in feeding (hypothalamus) and reward/reinforcement (including VTA and NAC) [2,40,41]. Recently, it was found that pretreatment with CB1R antagonist/rimonabant significantly reduced the ghrelin-induced activation of the mesolimbic dopamine system in mice [42]. Our previous studies in rat opioid addiction models discovered significant interactions between ghrelin and endocannabinoid (AEA, 2-AG) systems within the VTA and NAC (beside dopamine, opioid and GABA interactions) [43,44]. These results indicate potential interactions of ghrelin and cannabinoid signalling within reinforcement processes that also participate in addiction. 

So far, the role of ghrelin/GHS-R1A in the cannabinoid/CB1 agonist rewarding/reinforcing effects and addiction remains unclear. Our recent studies in rats (in preparation for this Special Issue) documented that ghrelin/GHS-R1A antagonist (JMV2959) significantly decreased THC-conditioned place preference (CPP) and behavioural stimulation and reduced WIN55,212-2 intravenous self-administration (IVSA) and tendency to relapse. The aim of the present study was to ascertain if the JMV2959 pretreatment could attenuate the cannabinoid-induced accumbens dopamine release and influence its metabolic turnover. WIN55,212-2 was administered into the posterior VTA, since the VTA is considered crucial for cannabinoid-induced mesolimbic dopamine activation [2,3,45]. Simultaneously, changes in endocannabinoids (i.e., AEA, 2-AG) and GABA in the NACSh were observed, considering complex reinforcing mechanisms of cannabinoids. Further, we tested if JMV2959 could also reduce systemic WIN55,212-2-induced behavioural stimulation, which is considered to be a sign of nigrostriatal dopaminergic pathway and contributes to drug addiction [46].

## 2. Results

### 2.1. Behavioural Testing in the LABORAS Cage

**Figure 1:**
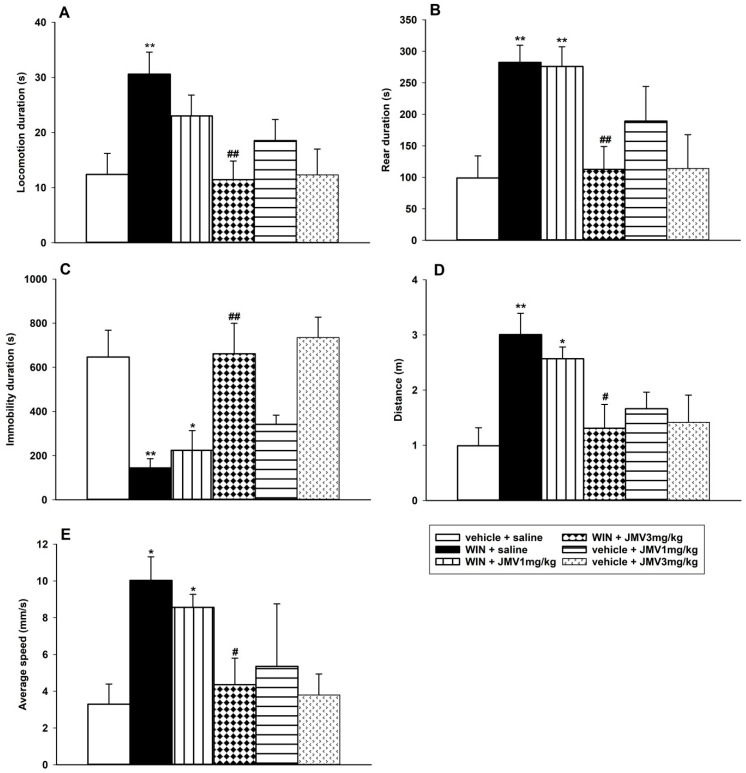
Effects of JMV2959 on the WIN55,212-2-induced behavioural changes in rats, in the fully automated behaviour monitoring LABORAS cage. JMV2959 (0, 1 and 3 mg/kg i.p.) was injected immediately before placing the rat into the cage, and after 20 min of habituation a stimulatory WIN55,212-2 dose 0.1 mg/kg or vehicle was administered intraperitoneally. After another 20 min of habituation, behaviour monitoring started and lasted for 20 min (20–40 min after WIN55,212-2 administration). Changes in locomotion duration (**A**), rear duration (**B**), immobility duration (**C**), distance travelled (**D**) and average speed overall (**E**) are illustrated as follows: saline + vehicle (open bar; *n* = 9), saline + WIN55,212-2 (filled bar; *n* = 7), JMV2959 1 mg/kg + WIN55,212-2 (vertically striped bar; *n* = 7), JMV2959 3 mg/kg + WIN55,212-2 (diamond bar; *n* = 8), JMV2959 1 mg/kg + vehicle (horizontally striped bar; *n* = 4), JMV2959 3 mg/kg + vehicle (little arrows bar; *n* = 8). The JMV2959 pretreatment effects in comparison to saline + WIN55,212-2 group are expressed as # *p* < 0.05, ## *p* < 0.01. Differences among groups in comparison to vehicle + saline group are expressed as * *p* < 0.05, ** *p* < 0.01.

Within 20–40 min after administration, the 0.1 mg/kg i.p. WIN55,212-2 dose produced significant behavioural stimulation in the habituated rats monitored using the fully automated LABORAS cage, in comparison to the vehicle + saline group (Figure 1). This is in accordance with the literature [47,48]. Following WIN55,212-2 administration, the locomotion, rear, distance travelled and average speed significantly increased, while immobility significantly decreased in comparison to the saline + vehicle group. The 1 or 3 mg/kg JMV2959 administered 20 min before WIN55,212-2, dose-dependently reduced the WIN55,212-2-induced changes in all monitored parameters using one-way ANOVA followed by the Holm–Shidak test; however, only pretreatments with the higher dose, 3 mg/kg JMV2959, reached significance in their effects; specifically: locomotion duration (F5,37 = 3.65, *p* = 0.009) (Figure 1A), rear duration (F5,37 = 5.88, *p* < 0.001) (Figure 1B), immobility duration (F5,37 = 5.72, *p* < 0.001) (Figure 1C), distance travelled (F5,37 = 4.22, *p* = 0.004) (Figure 1D) and average speed overall (F5,37 = 5.59, *p* < 0.001) (Figure 1E). Both doses of JMV2959 administered alone/with vehicle did not cause significant changes in rat behaviour during the monitored period in comparison to the vehicle + saline group.

### 2.2. In Vivo Microdialysis

The effects of 2.4 mM/0.5 µL WIN55,212-2 or 0.5 µL vehicle (Ringer solution with one drop of Tween 80) administration into the posterior VTA were monitored after pretreatment with JMV2959 3 mg/kg or saline injected intraperitoneally 20 min before the cannabinoid/vehicle. The NACSh microdialysis in vivo was used. The microdialysates raw data of the neurotransmitters/metabolites expressed as ng/mL were converted to baseline percentage levels (mean of three 20 min intervals prior to pretreatments), and these values were statistically analysed. The baseline microdialysate extracellular concentrations (ng/mL or pg/mL) obtained by the LC-MS analyses are summarized in Tables S1 and S2 in the Supplementary Materials. The extracellular ng/mL or pg/mL concentrations were used for calculation of the dopamine turnover metabolic ratios. 

#### 2.2.1. The JMV2959 Effects on the WIN55,212-2-Induced Increase of Dopamine and Its Metabolites in the NACSh

**Figure 2:**
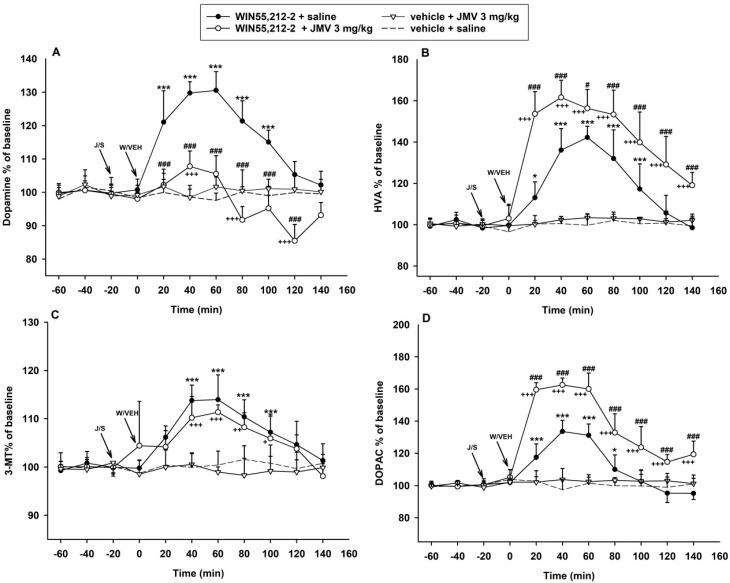
Effects of growth hormone secretagogoue receptors (GHS-R1A) antagonist (JMV2959) on WIN55,212-2-induced dopamine and its metabolites’ extracellular changes in the rat nucleus accumbens shell (NACSh). JMV2959 3 mg/kg was given i.p. 20 min before 2.4 mM/0.5 µL WIN55,212-2 or vehicle 0.5 µL was administered into the posterior ventral tegmental area (VTA) injection (*n* = 6; means ± SEM). Changes in accumbens dopamine are illustrated in graph (**A**), changes in homovanillic acid (HVA), 3-methoxytyramine (3-MT) and 3,4-dihydroxyphenylacetic acid (DOPAC) are illustrated in the graphs (**B**–**D**), respectively. The effects are illustrated as follows: saline + WIN55,212-2 (filled circle; *n* = 7), 3 mg/kg JMV2959 + WIN55,212-2 (open circle; *n* = 6), 3 mg/kg JMV2959 + vehicle (open triangle; *n* = 4), saline + vehicle (dotting; *n* = 7). Differences between saline + WIN55,212-2 and saline + vehicle groups are expressed as *** *p* < 0.001, * *p* < 0.05. Differences JMV2959 + WIN55,212-2 and saline + vehicle groups are expressed as +++ *p* < 0.001, ++ *p* < 0.01, + *p* < 0.05. Differences between groups saline + WIN55,212-2 and 3 mg/kg JMV2959 + WIN55,212-2 are expressed as ### *p* < 0.001, # *p* < 0.05.

The extracellular accumbens changes were observed for a total of 200 min (140 min after the cannabinoid application). The two-way ANOVA for repeated measures (RM) followed by Bonferroni’s test of accumbens microdialysis measurements of dopamine revealed an overall effect of the treatment (F3,8 = 125.3, *p* < 0.001), time (F8,168 = 22.1, *p* < 0.001) and the treatment × time interaction (F8,168 = 15.3, *p* < 0.001) (illustrated in Figure 2A). The rapid cannabinoid-induced dopamine increase in NACSh was significantly higher relatively to the vehicle + saline group during the 20–100 min intervals (*p* < 0.001). The maximal WIN55,212-2-induced dopamine increase (130.5% of baseline mean during the 60 min interval) was significantly reduced (by 22.7%) after the GHS-R1A antagonist/JMV2959 pretreatment to the maximum of 107.8% of the baseline mean (during the 40 min interval). However, despite the JMV2959 pretreatment, the cannabinoid-induced increase in accumbens dopamine remained significant relative to the vehicle + saline treatment at the time interval of 40 min (*p* < 0.001) and 60–80 min (*p* < 0.05). Subsequently, the dopamine extracellular concentration moderately dropped below baseline mean levels, oscillating from 95.2 to 91.7% and 85.4% of the baseline mean, respectively; the decrease was transiently significant relatively to the vehicle + saline group at intervals 80 min (*p* < 0.01), 100 min (*p* < 0.05) and 120 min (*p* < 0.001). Within the last 140 min interval, the dopamine levels in the JMV2959 + WIN55,212-2 group were comparable with the saline + WIN55,212-2 and saline + vehicle groups.

The effects of WIN55,212-2-induced dopamine metabolism in the NACSh are illustrated in the Figure 2B–D. The two-way ANOVA RM/Bonferroni analysis of accumbens 3-methoxytyramine (3-MT) measurements (Figure 2C) found an overall effect of the treatment (F3,8 = 18.0, *p* < 0.001), time (F8,168 = 12.2, *p* < 0.001) and the treatment × time reaction (F8,168 = 5.0, *p* < 0.001). The 3-MT accumbens extracellular levels were increased by the intra-tegmental WIN55,212-2 administration only to a maximum of 113.9% of the baseline mean (60 min interval). The JMV2959 pretreatment seemed to decrease the cannabinoid-induced 3-MT accumbens augmentation, but the effect was not significant with a maximum 111.3% of the baseline at a 60 min interval. 

The two-way ANOVA RM/Bonferroni test of extracellular concentration of 3,4-dihydroxyphenylacetic acid (DOPAC) in the NACSh, illustrated in Figure 2D, revealed an overall effect of the treatment (F3,8 = 102.3, *p* < 0.001), time (F8,168 = 53.4, *p* < 0.001) and the treatment × time reaction (F8,168 = 22.6, *p* < 0.001). Administration of WIN55,212-2 increased the extracellular accumbens DOPAC to a maximum of 133.5% of the baseline mean (40 min interval) and the DOPAC concentration was significantly higher relative to the vehicle + saline group during 20–60 min intervals (*p* < 0.001) and 80 min interval (*p* < 0.05). Pretreatment with JMV2959 enhanced the cannabinoid-induced DOPAC accumbens’ levels by 29% to a maximum of 162.5% of the baseline mean (40 min interval) and prolonged the significantly higher DOPAC levels relative to the vehicle + saline group until the end of the dialysis experiment (20–140 min interval, *p* < 0.001).

The statistical analysis of the accumbens extracellular concentrations of the final dopamine degradation metabolite, homovanillic acid (HVA), revealed an overall effect of the treatment (F3,8 = 52.4, *p* < 0.001), time (F8,168 = 61.8, *p* < 0.001) and treatment × time interaction (F8,168 = 21.8, *p* < 0.001) as illustrated in Figure 2B. The WIN55,212-2 increased accumbens HVA levels were significantly higher relatively to the vehicle + saline group within 40–100 min intervals (*p* < 0.001). Pretreatment with the GHS-R1A antagonist/JMV2959 significantly enhanced the maximal cannabinoid-increased accumbens HVA concentrations (142.2% of the baseline mean at a 60 min interval) by 19.4% to a maximal 161.6% of the baseline mean (at a 40 min interval). The HVA levels were significantly higher relatively to the vehicle + saline group from the first interval after the WIN55,212-2 administration and remained so until the end of the microdialysis trial (20–140 min intervals, *p* < 0.001).

A single 3 mg/kg intraperitoneal dose of JMV2959 + saline and also vehicle + saline had no significant effect on dopamine and its metabolites concentrations in the NACSh.

#### 2.2.2. The JMV2959 Effects on the WIN55,212-2-Induced Extracellular Turnover of Dopamine in the NACSh

We were interested in the possible effect of JMV2959 pretreatment on the cannabinoid-induced dopamine turnover in the NACSh. Therefore, we compared the dopamine turnover metabolic ratios of baseline levels (three 20 min intervals before pretreatments) with two intervals with the highest observed dopamine/metabolite changes induced by WIN55,212-2 administration, the 40 and 60 min intervals. We chose the groups WIN55,212-2 + saline, WIN55,212-2 + JMV2959 and vehicle + saline for comparison of the JMV2959 pretreatment effect. For analysis of dopamine turnover, the values of detected concentrations (pg or ng/mL) within the appropriate intervals were used. The average accumbens dopamine and metabolites concentrations (mean of the rat group) within the chosen intervals and rat groups are summarized in Table S1 in the Supplementary Materials. The dopamine turnover metabolic ratios were calculated as follows: for each animal baseline value of the metabolite concentration (−60, −40 and −20 min interval values) and the aftertreatment levels (+40 and +60 min interval values) were divided by the baseline concentration values (analogously −60, −40 and −20 min interval values) and after treatment (+40 and +60 min interval values) levels of dopamine. Before the calculation, the concentration data were all passed to pg/mL units. In this way, relative data were obtained (expressed as a mean ± SEM).

**Figure 3:**
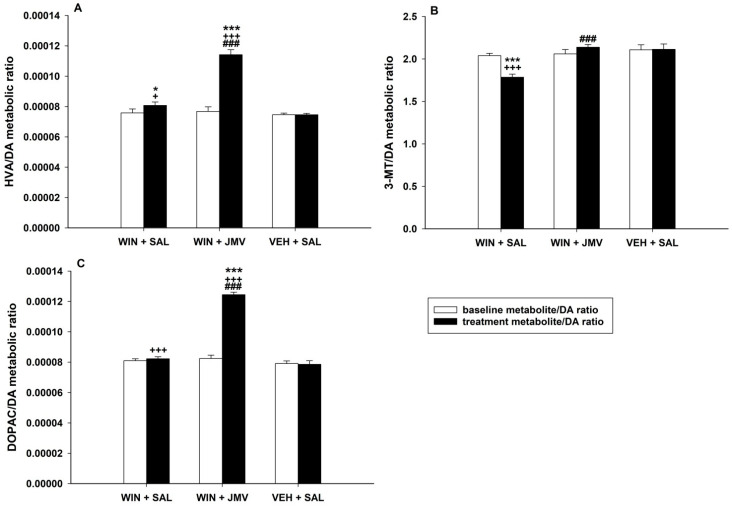
Extracellular dopamine (DA) metabolic turnover in the NACSh. The graphs show the metabolite/dopamine ratios, the means ± SEMs (*n* = 6) of concentration values of the metabolite divided by the corresponding values of dopamine concentrations (metabolite/DA). The homovanillic acid/dopamine ratio (HVA/DA) values are illustrated in the graph (**A**), 3-methoxytyramine/dopamine ratio (3-MT/DA) in the graph (**B**) and the 3,4-dihydroxyphenylacetic acid/dopamine ratio (DOPAC/DA) in the graph (**C**). Three baseline interval concentration values before pretreatments (baseline metabolite/DA ratio–white bars) and two intervals with maximal WIN55,212-2 effects (40 min and 60 min intervals; treatment metabolite/DA ratio–black bars) were used for statistical comparison within WIN55,212-2 + saline versus WIN55,212-2 + JMV2959 versus vehicle + saline groups. The effect of JMV2959 pretreatment, thus differences between WIN55,212-2 + saline and WIN55,212-2 + JMV2959, are expressed as ### *p* < 0.001. Differences between groups relative to the vehicle + saline group are expressed as + *p* < 0.05 and +++ *p* < 0.001. Differences between treatment and baseline are expressed as * *p* < 0.05 and *** *p* < 0.001.

Comparison of the dopamine (DA) turnover metabolic HVA/DA ratios, illustrated in Figure 3A, using two-way ANOVA/Bonferroni revealed significant difference among groups (F1,2 = 121.0; *p* < 0.001), treatment effect (F2,99 = 95.2; *p* < 0.001) and group × treatment interaction (F2,99 = 78.4; *p* < 0.001). These results indicate that total accumbens dopamine metabolism was significantly elevated after the WIN55,212-2 intracerebral administration, since its final metabolite (HVA) was increasingly produced; hence, the HVA/DA ratio was enhanced in comparison to the baseline as well as to the vehicle + saline group (*p* < 0.05). The JMV2959 pretreatment significantly increased the general accumbens dopamine turnover in comparison to saline pretreatment before WIN55,212-2, because, despite the observed accumbens dopamine decrease, the HVA concentration increased after the JMV2959 pretreatment together with the HVA/DA ratios (*p* < 0.001). Within the accumbens DOPAC/DA metabolic ratios (illustrated in Figure 3C), the two-way ANOVA/Bonferroni found significant differences among groups (F1,2 = 153.0; *p* < 0.001), treatment effect (F2,99 = 171,2; *p* < 0.001) and group × treatment interaction (F2,99 = 135.1; *p* < 0.001). Production of DOPAC in the NACSh after the cannabinoid administration was significantly increased only in comparison to the vehicle + saline group (*p* < 0.001). The JMV2959 pretreatment before WIN55,212-2 significantly elevated the accumbens DOPAC levels while reducing dopamine, so the DOPAC/DA ratios significantly increased relative to the WIN55,212-2 + saline group (*p* < 0.001). Comparison of the accumbens 3-MT/DA metabolic ratios using two-way ANOVA/Bonferroni revealed significant differences among groups (F1,2 = 4.3; *p* < 0.05), treatment (F2,99 = 18.5; *p* < 0.001) and group × treatment interaction (F2,99 = 12.5; *p* < 0.001). The dopamine efflux induced by WIN55,212-2 intracerebral administration in the NACSh was higher in comparison to the 3-MT levels production; thus, the 3-MT/DA ratios decreased relative to the baseline as well as to the vehicle + saline group (*p* < 0.001). The JMV2959 pretreatment almost prevented the WIN55,212-2-induced accumbens dopamine increase, yet the accumbens 3-MT production did not significantly change; thus, the 3-MT/DA ratios increased relative to the WIN55,212-2 + saline group (*p* < 0.001).

#### 2.2.3. The JMV2959 Effects on the WIN55,212-2-Induced Changes of Anandamide, 2-AG and GABA in the NACSh

**Figure 4:**
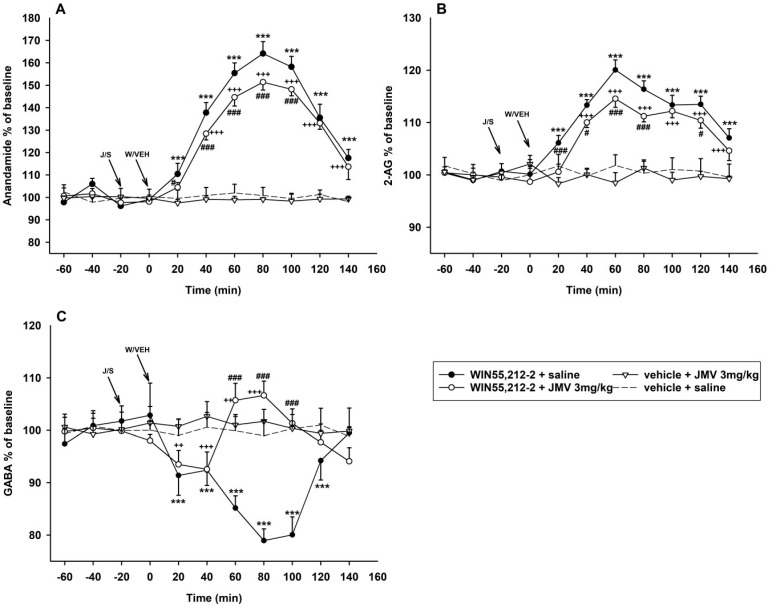
Effects of GHS-R1A antagonist (JMV2959) on WIN55,212-2-induced endocannabinoid and gamma-aminobutyric acid (GABA) extracellular changes in the rat NACSh. The JMV2959 3 mg/kg was injected i.p. 20 min before 2.4 mM/0.5 µL WIN55,212-2 or vehicle 0.5 µL administered into the posterior VTA injection (*n* = 6; means ± SEM). Changes in accumbens anandamide/N-arachidonoylethanolamine (AEA) levels are illustrated in graph (**A**), changes in 2-AG levels in the graph (**B**) and GABA changes are shown in graph (**C**). The effects are illustrated as follows: saline + WIN55,212-2 (filled circle; *n* = 7), 3 mg/kg JMV2959 + WIN55,212-2 (open circle; *n* = 6), 3 mg/kg JMV2959 + vehicle (open triangle; *n* = 4) and saline + vehicle (dotting; *n* = 7). Differences between saline + WIN55,212-2 and saline + vehicle groups are expressed as *** *p* < 0.001. Differences between JMV2959 + WIN55,212-2 and saline + vehicle groups are expressed as +++ *p* < 0.001, ++ *p* < 0.01. Differences between groups saline + WIN55,212-2 and 3 mg/kg JMV2959 + WIN55,212-2 are expressed as ### *p* < 0.001, # *p* < 0.05.

The measured microdialysate concentrations in pg/mL and ng/mL of anandamide/AEA, 2-AG and GABA in the baseline intervals and two intervals with maximal effects of WIN55,212-2/vehicle + JMV2959/saline (60 min, 80 min) are illustrated in Table S2 in Supplementary Materials. The two-way ANOVA RM/Bonferroni test of accumbens microdialysis measurements of N-arachidonoylethanolamine (anandamide, AEA) revealed an overall main effect of the treatment (F3,8 = 660.3; *p* < 0.001), time (F8,168 = 274.4; *p* < 0.001) and the treatment × time interaction (F8,168 = 97.5; *p* < 0.001) (see the Figure 4A). WIN55,212-2 administration into the VTA induced significant anandamide increase in the NACSh to a maximum of 164.1% of the baseline mean (80 min interval), and the AEA accumbens levels were significantly higher relatively to the vehicle + saline group at 20 min interval and remained so until the end of experiment (140 min interval) (*p* < 0.001). Pretreatment with JMV2959 transiently significantly reduced the cannabinoid-induced anandamide increase within a 20 min interval (*p* < 0.05) and 40–100 min intervals (*p* < 0.001) to a maximum of 151.4% of the baseline mean; thus, no more than by 12.7%. Despite the JMV2959 pretreatment, the cannabinoid-induced AEA increase remained significantly relative to the vehicle + saline group from the 40 min interval until the end of the experiment (140 min interval). 

Statistical analysis of accumbens 2-arachidonoylglycerol (2-AG) measurements revealed an overall main effect of the treatment (F3,8 = 182.5; *p* < 0.001), time (F8,168 = 79.8; *p* < 0.001) and the treatment × time interaction (F8,168 = 30.7; *p* < 0.001) (two-way ANOVA RM/Bonferroni) (see the Figure 4B). The accumbens 2-AG extracellular concentration was significantly increased by WIN55,212-2 administration from 20 min interval until the end of microdialysis (140 min interval) relatively to the vehicle + saline group (*p* < 0.001) with a maximum 120.1% of the baseline mean. The JMV2959 pretreatment transiently significantly reduced the cannabinoid-induced 2-AG elevation within 20 min and 60–80 min intervals (*p* < 0.001) and 40 min and 120 min intervals (*p* < 0.05) by 5.5% to a maximum of 114.5% of the baseline. Despite JMV2959 pretreatment, the WIN55,212-2-induced accumbens 2-AG elevation remained significant relative to the vehicle + saline group within 40–140 min intervals.

The two-way ANOVA RM/Bonferroni of accumbens γ-aminobutyric acid (GABA) microdialysis measurements revealed an overall main effect of the treatment (F3,8 = 52.4; *p* < 0.001), time (F8,168 = 8.5; *p* < 0.001) and the treatment × time interaction (F8,168 = 24.7; *p* < 0.001) (see the Figure 4C). The WIN55,212-2 administration into the VTA induced a significant decrease of accumbens GABA extracellular levels within 20–120 min intervals (*p* < 0.001) with a maximum of 78.9% of the baseline mean at an 80 min interval. When JMV2959 was injected i.p. 20 min before the cannabinoid, we observed an initial moderate diminution of GABA in the NACSh significant relatively to vehicle + saline group within 20 min and 40 min intervals (*p* < 0.01 and *p* < 0.001, respectively) with a maximum of 92.6% of the baseline. However, the drop in accumbens GABA was transformed into a mild but significant increase at the 60 min interval (*p* < 0.05) to a maximum of 106.6% of the baseline at the 80 min interval, and then the GABA levels decreased again back to baseline mean levels (non-significant relative to the vehicle + saline group). Thus, the WIN55,212-2 + saline induced 2-AG decrease in the NACSh was significantly changed/reversed by JMV2959 pretreatment within 60–100 min intervals (*p* < 0.001).

Single administration of 3 mg/kg JMV2959 i.p. or vehicle + saline did not induce significant changes of either AEA, 2-AG or GABA in the NACSh.

## 3. Discussion

The present study demonstrates that the central ghrelin signalling system, involving GHS-R1A, is required for indirect measures of the rewarding/reinforcing effects of the cannabinoid/CB1 agonist/WIN55,212-2 that participate in cannabinoid addiction. To our knowledge, our results, for the first time, document that GHS-R1A antagonism significantly reduced the cannabinoid-induced dopamine efflux in the nucleus accumbens shell (NACSh). A low dose of WIN55,212-2 (2.4 mM/0.5 µL) administered into the posterior ventral tegmental area provoked significant accumbens dopamine release together with extracellular endocannabinoids, anandamide/AEA and 2-AG, increase and transient GABA decrease, and all these observed cannabinoid-induced accumbens changes were significantly reduced by the GHS-R1A antagonist/JMV2959 3 mg/kg i.p. pretreatment 20 min before the cannabinoid. In addition, the WIN55,212-2-induced accumbens dopamine turnover was significantly increased by the JMV2959 pretreatment. Further, the behavioural stimulation observed within 20–40 min after the 0.1 mg/kg WIN55,212-2 intraperitoneal administration was significantly reduced by the JMV2959 3 mg/kg pretreatment. These results indicate substantial participation of ghrelin/GHS-R1A mechanisms in the rewarding/reinforcement effects of the CB1R agonist/synthetic cannabinoid WIN55,212-2, which are involved in cannabinoid addiction.

The cannabinoids/CB1R agonists, including WIN55,212-2, are known for their biphasic/dual effects, with low doses inducing the locomotor hyperactivity and with higher doses the hypoactivity. Behavioural stimulation, frequently observed in drugs of abuse, is considered to be a sign of dopaminergic nigrostriatal pathway activation and constitutes to part of the addiction process associated with drugs’ reinforcing effects, hence, contributing to drug dependence [46,47,48,49]. It was described that the low cannabinoid/WIN55,212-2 dose (0.1 mg/kg i.p.) increased the horizontal and vertical activity in habituated rats [47,48]. Also, in our experiment in the LABORAS cage, we observed a significant increase in locomotion, rearing, distance travelled and the overall average speed of behaviour in comparison to the vehicle treated group, within 20–40 min after WIN55,212-2/vehicle administration, when the initial explorative locomotor activity of rats declined and the advanced stimulatory effects of the cannabinoid were unmasked. The JMV2959 (1 and 3 mg/kg) administration 20 min before the cannabinoid (simultaneously starting the habituation period in the LABORAS cage), significantly reduced the monitored cannabinoid-stimulated behavioural parameters; however, only when the higher dose 3 mg/kg JMV2959 was used (*p* < 0.001). These results are in accordance with previous studies, when JMV2959 in mice or rats reduced locomotor hyperactivity induced by alcohol [50], nicotine [51], cocaine and amphetamine [52], morphine [53,54] and fentanyl [44]. When JMV2959 was administered alone (in the same doses), no significant influence on rat behaviour was observed within the relevant period in LABORAS, which corresponds well with our former study in activity cage/open field (these results are summarized in Table S3 in the Supplementary Materials) [55]. 

Cannabinoids including the main cannabis constituent tetrahydrocannabinol (THC) and synthetic cannabinoids (e.g., WIN55,212-2) most likely mediate their pleasurable, anxiolytic and rewarding/reinforcing effects through the CB1Rs located within the central brain reward circuits, particularly the VTA and the NAC [3,10,11]. Cannabinoids activate these reward pathways in a manner that is consistent with other drugs of abuse, including triggering dopamine release in the nucleus accumbens shell (NACSh) [1,2,3,19,23,25,56]; however, particular mechanisms through which this occurs seem to be more specific; it would appear that THC (and probably other exogenous cannabinoids) may have somewhat pathway-dependent effects in this brain reward circuit (further research is needed) [57,58]. The cannabinoid mechanisms of effect and the endocannabinoid system involvement are described in more detail in the Supplementary Materials.

Research studies that used cannabinoid experimental addiction models discovered high dose-, strain/genetic-, drug- and brain substructure-sensitivity/dependence that can crucially influence the obtained results (e.g., see reviews [22,26,56]. The reinforcing effects shift rapidly to aversive effects with increasing dose [2,3]. Systemic cannabinoid/THC/WIN55,212-2-induced CB1R-dependent dopamine release was observed specifically in the NACSh and varied depending on the strain of rats assessed [23,25,59]. The THC was intra-cerebrally self-administered in rats, specifically into the posterior VTA and the NAC shell substructures but not into the anterior VTA or the NAC core [45,56]. Likewise, THC administration into the anterior VTA did not produce any dopamine release in rat NACSh (in vivo microdialysis) [60].

In accordance with the findings mentioned above and in the Supplementary Materials, in our in vivo microdialysis study, local administration of the low 2.4 mM/ 0.5 µL dose of WIN55,212-2 into the posterior VTA induced significant dopamine efflux in the NACSh of Wistar rats [1,2,3,19,45,48]. The intracerebral 2.4 mM dose of WIN55,212-2 induced dopamine release with a maximum of 131% of the baseline mean, which approximately corresponds with published microdialysis experiments when WIN55,212-2 was administered systemically (i.p., i.v.) and the dopamine release was prevented by CB1 antagonist (rimonabant) co-administration [25,48]. 

The intra-VTA administration of WIN55,212-2 also increased accumbens extracellular concentrations of dopamine degradation metabolites: 3-methoxytyramine/3-MT (a maximum of 114% of the baseline mean), 3,4-dihydroxyphenylacetic acid/DOPAC (133%) and homovanillic acid/HVA (142% of the baseline mean). The maximal microdialysate concentration values (40 and 60 min intervals after WIN55,212-2 administration) were used for calculation of the extracellular dopamine metabolic turnover ratios. The cannabinoid application significantly increased the extracellular dopamine metabolic turnover HVA/DA ratio (*p* < 0.05) in comparison with the control/vehicle + saline group. The 3-MT/DA ratio significantly decreased (*p* < 0.001) and the DOPAC/DA ratio increased (*p* < 0.001). Increases in dopamine metabolism, measured with the DOPAC/DA ratio, have been reported in most but not all rodent studies with cannabinoids [19,61,62]. Some resulting inconsistencies within the early ex-vivo studies could be explained due to the technical limitations in detecting the rapid changes in extracellular dopamine/metabolite concentration, detectable by microdialysis techniques used more recently [19]. 

Pretreatment with the GHS-R1A antagonist/JMV2959 3 mg/kg i.p. 20 min before WIN55,212-2 significantly reduced the cannabinoid-induced dopamine efflux in the NACSh. The same 3 mg/kg i.p. JMV2959 dose “per se” did not influence accumbens dopamine, as it has already been described [50,53,63]. Also, the lower JMV2959 dose 1 mg/kg alone did not induce significant changes in accumbens dopamine/metabolites (see Figure S1 in the Supplementary Materirals). The observed GHS-R1A antagonist effect is in accordance with previous studies in mice/rats experimental models with alcohol [50], stimulants/cocaine, amphetamine [52], nicotine [51] and opioids/morphine and fentanyl [53,54,63]. Contrary to alcohol and stimulant models, the JMV2959 pretreatment did not completely abolish the cannabinoid-induced accumbens dopamine increase. The dopamine extracellular concentrations remained transiently significantly increased (108% of the baseline mean) in comparison with baseline and the vehicle + saline group, similar to studies with opioids, when the JMV2959 pretreatment effects were weaker [53,54,63]. However, in the present study, the JMV2959 + WIN55,212-2-induced transient dopamine increase (40–80 min intervals) was changed to transient moderate but significant decrease (in the 120 min interval; maximum of 86% of the baseline mean, *p* < 0.001), which was not observed in previous studies. The observed gradually intensified JMV2959 pretreatment effect could indicate some modulatory mechanisms of the GHS-R1A antagonism specifically in combination with the cannabinoid. However, in contrast to previous studies, when the drugs were administered systemically (s.c. or i.v.), in this case the cannabinoid was injected directly into the VTA, which also may impact the effect proportions. Nevertheless, the GHS-R1A antagonism significantly reduced the cannabinoid/CB1R-induced accumbens dopamine efflux, the trigger of reinforcement effects, which indicates the important involvement of the ghrelin/GHS-R1A system in the cannabinoid reinforcement effects, hence, addiction processing.

The appropriate specific involved mechanisms have to be further researched. The CB1Rs are located on various presynaptic axons/inputs in the VTA and the NAC [3,10,11]. Concerning the GHS-R1As, it was described that intra-VTA administered ghrelin induced accumbens dopamine release and the intra-VTA GHS-R1A antagonist supressed this effect [37,64]. The GHS-R1A is expressed on dopaminergic neurons [29], and it has been suggested that GHS-R1A regulates the activity of VTA dopamine neurons via heterodimerization of the GHS-R1A to the D1R (especially with stimulants), as well as by the strong constitutive activity of the GHS-R1A [31,65]. Further, the midbrain GHS-R1As, co-localized with dopaminergic and cholinergic receptors [35,36], activate the cholinergic–dopaminergic reward link and trigger the VTA dopaminergic signalling, which can be blocked by intra-VTA administration of nicotinic cholinergic and glutamatergic receptor antagonists [37,38,66]. Also, GHS-R1A is known to modulate synaptic plasticity in the brain [67,68]. The GHS-R1As are also expressed on the GABA VTA neurones [67] however, the ghrelin-induced activation of VTA dopamine neurons was not blocked in presence of GABA-A antagonist [39]. The CB1Rs and the GHS-R1As are evenly expressed in both the VTA and the NAC and also in hypothalamus. Substantial functional cooperation between the ghrelin and cannabinoid systems in regulation of food intake has been mentioned above [27,28,29,30,67]. Dimerization between CB1R and GHS-R1A has not been directly examined yet, but it was suggested [34]. In the hypothalamus, the GHS-R1A is present at GABAergic presynaptic terminals, and it attenuates GABA release in the hypothalamic neurons [69]. CB1-dependent long-term depression (LTD) in the VTA GABA neurons induced by cannabinoid/THC has been described [70]. The existing findings propose several possible modes of particular ghrelin/cannabinoid interaction mechanisms (e.g., on GABA axons or possibly on glutamate or cholinergic transmission (what we did not test)); however, further research is necessary. 

The accumbens extracellular dopamine metabolic turnover increase, induced by intracerebral WIN55,212-2 during its maximal effect (40 and 60 min intervals) in the present study, was significantly augmented after JMV2959 pretreatment in comparison to the WIN55,212-2 + saline group, measured with metabolic ratios HVA/DA, DOPAC/DA and 3-MT/DA (*p* < 0.001). The substantial increase in final metabolite HVA formation (HVA/DA ratio) was produced by enhanced DOPAC production (DOPAC/DA ratio). This is in accordance with our previous opioid microdialysis studies when a significantly increased formation of DOPAC and HVA in the NACSh after JMV2959 pretreatment before morphine and fentanyl was observed [54,63]. Thus, a significant impact of JMV2959 pretreatment was observed on cannabinoid- and opioid-provoked accumbens dopamine metabolism due to the monoamine oxidase (MAO) (the significant DOPAC and DOPAC/DA increase). Moreover, the relatively high 6 mg/kg i.p. JMV2959 dose, when administered alone, moderately but significantly increased DOPAC and HVA accumbens levels in rats [54]; we did not find any significant influence of single JMV2959 3 mg/kg on the accumbens dopamine/metabolite concentrations [54,63]. Altogether, it seems that GHS-R1A antagonism might be associated with increased metabolism of dopamine by MAO.

Simultaneously with the dopamine release, administration of WIN55,212-2 into the posterior VTA also induced the increased extracellular concentrations of anandamide and 2-AG in the NACSh. The maximum effects were observed in the same period as with dopamine efflux (2-AG, maximum of 120% of the baseline mean in the 60 min interval) or 20 min later (AEA, maximum of 164% of the baseline mean in the 80 min interval). The rapid extracellular endocannabinoids increase in the NACSh induced by the CB1R agonist-provoked dopamine release (possibly modulated by other factors) was expected in accordance with the in-detail described mechanisms in the Supplementary Material [3,19,58]. 

The 3 mg/kg i.p. JMV2959 pretreatment moderately but significantly reduced the cannabinoid-induced accumbens AEA increase (by a maximum of 12.7% of the baseline mean) as well as the 2-AG elevation (by a maximum of 5.5% of the baseline mean), but the endocannabinoid concentrations remained significantly higher relatively to the vehicle + saline group from the 40 min interval until the end of microdialysis (140 min interval). As in previous studies, the 3 mg/kg i.p. JMV2959 dose did not influence the accumbens AEA or 2-AG concentrations when it was administered alone [43,44]. The JMV2959 effect on the WIN55,212-2-induced accumbens dopamine release (described above) seems more pronounced than its influence of endocannabinoids. In our previous studies with opioids, we observed a massive effect of the ghrelin antagonist on fentanyl/morphine-induced accumbens AEA increase, which was reversed to decrease due to the JMV2959 pretreatment (administered intraperitoneally as well as into the VTA and the NACh). On the contrary, the opioid-induced 2-AG decrease in the NACSh was intensified by the JMV2959 pretreatment (with all mentioned administration modes) [44,63]. However, cannabinoids and opioids influence diversely the endocannabinoid modulations within the NAC [40,41]. 

In the present study, the cannabinoid-induced increase in accumbens AEA/2-AG levels were reduced moderately but consistently by the JMV2959 pretreatment and several mechanisms can be considered. The JMV2959 pretreatment significantly reduced the accumbens dopamine efflux, an important trigger of both endocannabinoids accumbens formation and release (from the MSNs or FSI as described in the Supplementary Materials) [3,58]. Further, the GHS-R1As are expressed within the NAC (e.g., on dopaminergic and also GABAergic neurons) [35,39]; thus, their antagonism could affect the relevant neural sensitivity, including the dopamine-independent mechanisms. The strong constitutive activity of the GHS-R1A should be also considered [31], although, the GHS-R1A inverse agonist would play a more important role. The dimerizations of GHS-R1A with D1R and D2R has already been confirmed [34]. Endocannabinoids are presumably produced by both accumbens iMSNs (D2R) as well as dMSNs (D1R), but most important endocannabinoid retrograde modulations were identified in the iMSN synapses [3,58]. We can only consider AEA/2-AG accumbens extracellular concentrations, because the consequent modulations were not investigated in our study. The GHS-R1A/D1R dimerization generally amplifies the D1R signalling [34,65]; thus, the GHS-R1A antagonist would reduce it. One of the possible consequences might be lower dMSN endocannabinoid production. The presence of ghrelin (GHS-R1A/D2R dimerization) appears to switch the D2R-intracellular signalling cascade [71]; therefore, the GHS-R1A antagonist would possibly attenuate this effect, so impact on AEA/2-AG production by iMSNs is possible but unclear. Moreover, the glutamate and acetylcholine involving interactions might participate in the endocannabinoid changes, which were not studied. Consequently, it seems that the GHS-R1A antagonism reduces the endocannabinoids modulatory effects induced by CB1R agonist in the NACSh due to the fact of their decreased availability, but further research is necessary to clarify the antagonists’ particular involvements. 

The WIN55,212-2 intra-VTA administration also induced a significant decrease in the γ-aminobutyric acid/GABA extracellular concentrations in the NACSh (maximum of 79% of the baseline in an 80 min interval), which was normalised within the last 140 min interval. The accumbens GABA decrease was presumably a consequence of the endocannabinoid presynaptic modulations (DSI and LTD) within the NAC, where CB1Rs are located on both the MSN collaterals and on intrinsic GABA neuron terminals, just as in the VTA [20,70]. The CB1R agonists (CP55940, THC) were found to decrease the accumbens GABA release [20,72]. It was suggested that the (endo)cannabinoid-induced inhibition of GABA release onto the MSNs in the NAC might increase/cause the disinhibition effect of the GABAergic output to the VTA and thus further participate on the cannabinoid reinforcement effects [20]. 

The JMV2959 pretreatment reversed the cannabinoid-induced accumbens GABA decrease to transiently significant increase (maximum of 107% of the baseline mean in the 80 min interval), which again was normalised within the last 140 min interval. The single 3 mg/kg JMV2959 dose did not significantly influence the accumbens GABA levels as in previous experiments [44]. In our study with opioids, the fentanyl-induced accumbens GABA enhancement was significantly reduced by the JMV2959 pretreatment [44]; however, accumbens GABA levels are influenced by opioids and cannabinoids in different ways [73,74]. The GHS-R1As are expressed on GABA neurons in the VTA, the NAC and elsewhere in the brain [67]. Important interactions between ghrelin and GABA systems have been implicated within the CeA [75] and the hypothalamus [69]. The GHS-R1As are located on the hypothalamic GABAergic presynaptic terminals; the GHS-R1A constitutive activity itself and administration of the GHS-R1A agonist attenuated GABA release. Presumably, the GHS-R1A antagonist and/or inverse agonist would reduce/abolish this effect. Similar interaction can be presumed within the NAC/VTA [67]. It seems that ghrelin and endocannabinoids show similar synaptic plasticity mechanisms, so they might cooperate on GABAergic neurons, where they are both located on the presynaptic axons; moreover, the CB1/GHS-R1A dimers were indicated [34]. Further investigation is necessary for clarifying the particular involved mechanism. 

It should also be mentioned that literary data suggested that an intact ghrelin signalling pathway is necessary for cannabinoid-induced food intake. Administration of CB1R agonist in the GHS-R1A knockout mice did not influence the food intake contrary to the wild/control mice, which showed a trend to increase food consumption with higher doses of HU210 injection [30]. In our studies, the food was removed after the drug administrations during the experiments; thus, we could not observe the single JMV2959 dose effects on food intake. No differences were observed in daily food consumption among the rat groups. 

Collectively, our results demonstrate that the GHS-R1 antagonist significantly reduced the CB1R agonist/WIN55,212-2-induced behavioural stimulation, the dopamine and endocannabinoid (AEA, 2-AG) release in the NACSh and reversed the accumbens GABA decrease. Also, it simultaneously increased the extracellular dopamine accumbens metabolism through MAO. In our further recent study (in preparation for this Special Issue), we have found that the same GHS-R1A antagonist (JMV2959) significantly reduced the WIN55,212-2 intravenous self-administration (IVSA) and tendency to relapse, the THC-induced condition place preference (CPP) development as well as expression and reduced the THC-induced behavioural stimulation. Altogether, our results suggest substantial involvement of ghrelin/GHS-R1A central signalling in the cannabinoid rewarding/reinforcement pro-addictive effects, which encourages further investigation of the GHS-R1A antagonism as a potential approach to cannabinoid addiction treatment.

## 4. Materials and Methods

### 4.1. Animals

Male adult Wistar rats (Velaz, Prague, Czech Republic) initially aged 8 weeks were used in all the experiments. The number of rats in the experimental groups within the behavioural LABORAS study were 9–4, and in the microdialysis study 7–4. At least seven days before the start of the experiment and at the end of each trial, the rats were given free access to food and water and were housed in polycarbonate cages in threes (LABORAS) or singles (microdialysis) per cage with constant humidity (50–60%), room temperature (22–24 °C) and reversed 12 h light/dark cycle (6 a.m.–6 p.m.). Procedures involving animals, along with animal care, were conducted in accordance with international laws; protocols complied with the Guidelines of the European Union Council (86/609/EU, 24 November 1986), the EU Directive (2010/63/EU, 22 September 2010) and the instructions of the National Committee for the Care and Use of Laboratory Animals. Experiments were approved by the Expert Committee for Protection of Experimental Animals of the Third Faculty of Medicine, Charles University, Prague, and they were performed in accordance with the Animal Protection Act of the Czech Republic (No. 246/1992 Sb, 15 April 1992).

### 4.2. Drugs and Chemicals

The synthetic aminoalkylindole cannabinoid WIN 55,212-2 mesylate salt (WIN55,212-2) was provided by Sigma–Aldrich (Prague, Czech Republic). The GHS-R1A antagonist, substance JMV2959 (1,2,4-triazole derivate), was synthesized at the University of Chemistry and Technology Prague (UCT Prague, Czech Republic). WIN55,212-2 was firstly dissolved in one drop of Polysorbate 80 (Tween 80) and then mixed in saline or Ringers solution. Saline/Ringers solution with drop of Tween 8 (vehicle) and saline were used as a placebo/control. In accordance with the literature [48,62], WIN55,212-2 was administered intraperitoneally (i.p.) (in LABORAS) in a stimulatory dose of 0.1 mg/kg in volumes of 0.1 mL/100 g of body weight and intracerebrally (in microdialysis) into the posterior ventral tegmental area (VTA) in dose 2.4 mM/0.5 µL for one minute dissolved in Ringers solution (pH 7.0); the intra-cerebral cannula stayed in place for another minute and after was retracted (5μL microsyringe; Innovative Labor System, Stutzerbach, Germany). The selected doses of JMV2959 (1, 3 or 6 mg/kg) were determined based on our previous studies in Wistar rats [44,54,55,63]. The JMV2959 doses had no significant effect on the rat locomotor behaviour during the monitored time (from 20 min after administration) [55]. The JMV2959 was administered i.p. at 0.1 mL/100 g of body weight, always 20 min prior to the WIN55,212-2/vehicle administration. 

### 4.3. Behavioural Testing in LABORAS Cage

The LABORAS apparatus (Metris B.V., Netherlands) was used for testing the behavioural changes induced by the WIN55,212-2/vehicle with a JMV2959/saline pretreatment from 8 am to 16 pm in reversed light/dark cycle (during the dark period). The LABORAS is known as a fully automated system for monitoring of the behavioural postures and tracking of small rodents [76]. Immediately after the i.p. injection of saline or JMV2959 (1 or 3 mg/kg), rats were placed into the LABORAS cage for habituation; 20 min later, 0.1 mg/kg WIN55,212-2 or vehicle was administered i.p. and the rats were left in the cage for another 20 min for habituation. Then, the 20 min monitoring period started, within 20–40 min after WIN55,212-2 administration. Following the literature, this interval matched a period when significant 0.1 mg/kg WIN55,212-2-induced behavioural stimulation could be observed [47]. The following parameters were automatically evaluated by LABORAS: time spent in locomotion (s), time spent immobile (s), time spent rearing (s), time spent grooming [s], distance (trajectory length) (m), and average speed (mm/s). The animals were randomly assigned to groups. The vehicle + saline group served as a control to compare the effects of THC and the pretreatments.

### 4.4. In Vivo Microdialysis 

Acute effects of WIN55,212-2 after pretreatment with JMV2959 (3 mg/kg) or saline were monitored in rats using in vivo microdialysis of the nucleus accumbens shell (NACSh). Treatment groups were as follows: saline + vehicle; saline + WIN55,212-2 2.4 mM/0.5 μL; JMV2959 3 mg/kg + WIN55,212-2 2.4 mM/0.5 μL; JMV2959 3 mg/kg + vehicle. The dialysis samples were collected at 20 min intervals for a total of 200 min. Dialysates were analysed for the concentration of dopamine and its metabolites (3-methoxytyramine (3-MT), 3,4-dihydroxyphenylacetic acid (DOPAC) and homovanillic acid (HVA)) as well as endocannabinoids (anandamide/arachidonoylethanolamid/AEA and 2-arachidonoylglycerol/2-AG) and gamma-aminobutyric acid (GABA), using high-sensitivity liquid chromatography combined with mass spectrometry. 

**Figure 5:**
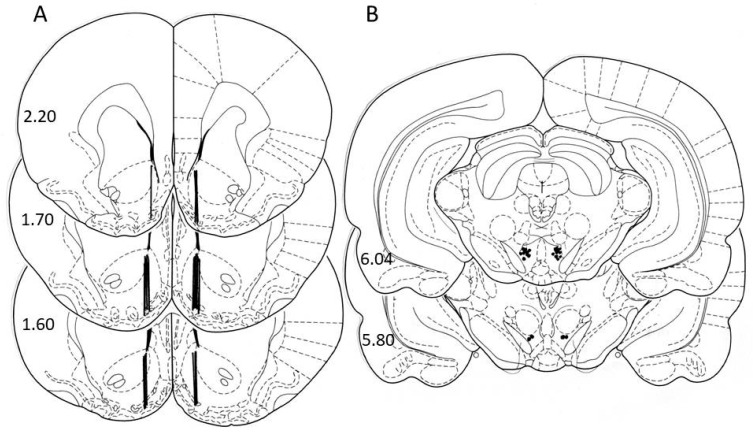
Schematic locations of dialysis probes in the nucleus accumbens shell (**A**); sites of infusions into the ventral tegmental area (**B**). Schematic locations of probe tips in animals which were involved in statistical analyses of accumbens neurotransmitter concentrations (the bold lines indicate the dialyzing positions in Figure 4A and locations of WIN55,212-2/vehiculum solution administrations into the VTA (dark dots in the lower part of slices in Figure 4B) as described in the atlas of Paxinos and Watson [77]. The distance from bregma (in mm) is indicated on the left of each sectional view.

The surgery under ketamine/xylazine anaesthesia was described in detail in our previous studies [43,44,63]. Two guide cannulas were randomly and unilaterally implanted, one into the NACSh (A: +2.0 mm and L: ±1.2 mm from bregma and V: 6.2 mm from occipital bone) and one into the posterior VTA (P: 6.0 mm and L: ±1.0 mm from bregma and V: 8.0 mm from the skull) [77]. The selected coordinates to target the VTA district that participate in the ghrelin as well as cannabinoid food/drug motivation processes were chosen following literature [39,45,78]. Postoperative, the rats were housed in their own individual cages. After the end of microdialysis experiments, the placements of the dialysis probe (NACSh) as well as the infusion cannula (VTA) were verified histologically (Figure 5). Only animals with a correct probe/cannula placement were used for statistical analysis. 

In accordance with References [44,63], 48 h after implantation, a probe (MAB4, 2 mm active cuprophane membrane, Agnthos, Sweden) was inserted into the guide cannula and was flushed with Ringer’s solution (pH 7.0) at a constant rate of 2.0 μL/min (Univentor 864 Syringe Pump, Agnthos, Sweden). After minimum 60 min of habituation, 20 μL samples were collected at 20 min intervals in small polyethylene tubes containing 7 µL HCl 0.1 mM to prevent monoamine degradation. The further 20 μL dialysate samples of each 20 min interval were collected in empty small polyethylene tubes for detection of the other neurotransmitters (endocannabinoids and GABA). After three consecutive baseline samples, the rats were injected with saline or 3 mg/kg JMV2959 (i.p.), which was followed (20 min later) by WIN55,212-2 (2.4 mM/0.5 μL) or vehicle solution administration into the VTA for one minute and the intra-cerebral cannula stayed in place for another minute and after was retracted. Sampling continued for 140 min following WIN55,212-2/vehicle. Immediately after sampling, the samples were frozen at −70 °C. 

The amount of the appropriate neurotransmitters/their metabolites in the dialysates were quantified using liquid chromatography combined with electrospray ionization tandem mass spectrometry (LC-ESI-MS/MS) (Thermo Scientific, Waltham, MA, USA), the method was previously described [43,44,54,79]. The in vitro recovery (probe MAB4, 2 mm, Agnthos, Sweden) of anandamide/AEA and 2-AG has been determined in our previous study [43]. The average recovery of AEA was 51 ± 4% and for 2-AG 53 ± 5%. The detected extracellular concentrations from the NACSh microdialysates oscillated around 1.9–3.9 ng/mL of AEA and 0.4–0.7 ng/mL of 2-AG. The limit of quantification (LOQ) for AEA was 240 pg/mL and the LOQ for 2-AG was 280 pg/mL. In our previous study, it was described that GABA efflux in the NACSh in our microdialysis experiments was Ca2+-dependent, which indicates that GABA extracellular concentration in the microdialysates reflects the overflow of neuronally released neurotransmitter (from the synapses) [44].

For analysing the JMV2959 pretreatment effects on the cannabinoid-induced dopamine turnover in the NACSh, the values of detected dopamine/metabolite concentrations (pg or ng/mL) were used, which were obtained within the appropriate intervals. The concentration data were all converted to pg/mL units. The dopamine turnover metabolic ratios were calculated as follows: for each animal the baseline value of the metabolite concentration (−60, −40 and −20 min interval values) and the aftertreatment levels (+40 and +60 min interval values) were divided by baseline concentration values (analogously −60, −40 and−20 min interval values) and after treatment (+40 and +60 min interval values) levels of dopamine. We compared these values only among WIN55,212-2 + saline versus WIN55,212-2 + JMV2959 versus vehicle + saline groups.

### 4.5. Statistical Analysis

Sigma Plot 13 (Systat Software, Inc., San Jose, CA, USA) was used for the statistical evaluation of the data. The behavioural changes among the rat groups with different treatments observed in the LABORAS cage within 20–40 min after the WIN55,212-2/vehicle administration, were evaluated by one-way ANOVA followed by the Holm–Sidak post–hoc test. The microdialysates raw data for dopamine and its metabolites, endocannabinoids and GABA expressed as ng/mL were converted to percentage of baseline levels (mean of three 20 min intervals prior to pretreatments). For statistical differences between the treatment groups relative to time-related changes during the in vivo microdialysis experiment, a two-way ANOVA RM/Bonferroni test was used. For statistical analysis of dopamine turnover metabolic ratios (comparison among the groups) a two-way ANOVA/Bonferroni method was used. All results are presented as a group mean ± SEM. All statistical tests were evaluated at a significance level of 0.05 (*p* values of < 0.05, < 0.01, and < 0.001 defined statistical significance). 

## Data Availability

The data presented in this study are available in the article and Supplementary Information.

## Supplementary Materials

Supplementary materials can be found at https://www.mdpi.com/1422-0067/22/1/210/s1.
