# Supplementary material for: Alterations in Rat Accumbens Dopamine, Endocannabinoids and GABA Content During WIN55,212-2 Treatment: The Role of Ghrelin
Source: Int J Mol Sci. 2020 Dec 28;22(1):210. doi: 10.3390/ijms22010210 (PMC7795825; doi:10.3390/ijms22010210)
Supplement: Supplementary file 1 [file ijms-22-00210-s001.pdf]

SUPPLEMENTARY INFORMATION

Results

2.2. In vivo microdialysis

2.2.2. The JMV2959 effects on the WIN55,212-2 –induced extracellular turnover of dopamine in the NACSh

The average accumbens concentrations (means within the group) of dopamine and its metabolites 3-MT, DOPAC and HVA within the three baseline intervals and the 40 min and 60 min intervals after the WIN55,212-2 administration, when maximum effects were observed (each microdialysis sampling interval lasted 20 min) are illustrated in Table S1. We were interested in the WIN55,212-2 effects and particularly the JMV2959 pretreatment effects on the cannabinoid-induced accumbens changes. Therefore, the follow groups were chosen for comparison: WIN55,212-2 + saline, WIN55,212-2 + JMV2959, vehicle + saline. Single concentration values from these rat groups and intervals were used for calculation of the particular accumbens metabolite/dopamine metabolic ratios (HVA/DA, 3-MT/DA, DOPAC/DA) (see the main text of article).

**dopamine metabolite**	**rat group**	**mean baseline values**			**mean after treatment values**	
		**bas -60 min**	**bas -40 min**	**bas -20 min**	**+40 min**	**+60 min**
**DOPAMINE (pg/ml)**	WIN+SAL	123.3±2.1	124.7±2.4	123.5±1.9	160.8±3.4	161.7±3.7
	WIN+JMV	127.0±3.2	127.9±3.2	126.4±2.3	136.7±1.8	133.9±2.1
	VEH+SAL	124.7±2.2	128.6±2.1	124.8±2.7	124.1±2.0	128.0±2.3
**HVA (ng/ml)**	WIN+SAL	9.3±0.3	9.6±0.3	9.2±0.3	12.7±0.3	13.3±0.2
	WIN+JMV	9.7±0.1	9.8±0.2	9.7±0.3	15.7±0.4	15.2±0.4
	VEH+SAL	9.4±0.2	9.4±0.1	9.3±0.2	9.4±0.1	9.3±0.1
**3-MT (pg/ml)**	WIN+SAL	250.3±2.0	254.3±2.2	252.0±2.2	286.9±2.7	287.3±5.1
	WIN+JMV	261.2±6.1	261.2±5.4	260.8±4.5	287.5±6.0	290.8±6.0
	VEH+SAL	262.1±4.2	261.1±4.0	262.1±4.9	261.5±4.6	263.0±5.9
**DOPAC (ng/ml)**	WIN+SAL	10.0±0.2	10.0±0.2	10.1±0.2	13.3±0.3	13.1±0.2
	WIN+JMV	10.5±0.2	10.4±0.2	10.5±0.2	17.0±0.3	16.7±0.5
	VEH+SAL	9.9±0.2	9.9±0.2	10.0±0.2	9.7±0.3	10.0±0.2

Table S1. The average accumbens concentrations (means within rat group) of dopamine and its metabolites 3-MT, DOPAC and HVA within three baseline intervals and the 40 min and 60 min intervals after the WIN55,212-2 administration, when maximum effects were observed (each sampling interval lasted 20 min). Number of rats in the groups: N = 7 (WIN55,212-2 +SALINE), N = 6 (WIN55,212-2 + JMV2959), N = 7 (VEHICLE + SALINE).

2.2.3. The JMV2959 effects on the WIN55,212-2 –induced changes of anandamid, 2-AG and GABA in the NACSh

The measured microdialysate concentrations (mean within the group) of anandamide/AEA, 2-AG and GABA in the baseline intervals and the two intervals with maximal effects of WIN55,212-2/vehicle + JMV2959/saline (60 min, 80 min) are illustrated in Table S2.

endocannabinoids GABA	rat group	mean baseline values			mean after treatment values	
		bas -60 min	bas -40 min	bas -20 min	+60 min	+80 min
AEA (ng/ml)	WIN+SAL	2.21±0.02	2.39±0.02	2.17±0.04	3.51±0.04	3.70±0.05
	WIN+JMV	2.30±0.05	2.32±0.03	2.26±0.02	3.30±0.02	3.46±0.02
	VEH+SAL	2.24±0.05	2.14±0.05	2.18±0.06	2.23±0.05	2.20±0.05
2-AG (pg/ml)	WIN+SAL	450.9±2.3	444.5±2.9	452.4±3.8	539.4±2.4	522.7±2.2
	WIN+JMV	456.4±1.5	454.5±2.2	452.6±3.6	520.6±2.5	505.4±1.9
	VEH+SAL	458.1±4.8	451.0±3.3	445.2±3.2	458.3±4.0	451.5±3.0
GABA (ng/ml)	WIN+SAL	5.25±0.08	5.43±0.06	5.48±0.08	4.58±0.04	4.25±0.06
	WIN+JMV	5.45±0.06	5.49±0.09	5.46±0.09	5.78±0.07	5.83±0.08
	VEH+SAL	5.34±0.08	5.42±0.07	5.37±0.08	5.37±0.09	5.32±0.06

Table S2. The average accumbens concentrations (means within rat group) of anandamide/AEA, 2-AG and GABA within the three baseline intervals and the 60 min and 80 min intervals after the WIN55,212-2 administration, once the maximum effects were observed (each sampling interval lasted 20 min). Number of rats in groups: N = 7 (WIN55,212-2 +SALINE), N = 6 (WIN55,212-2 + JMV2959), N = 7 (VEHICLE + SALINE).

Discussion

Table S3. summarises selected results from our previous behavioural study (Jerabek et al. 2017) which support/confirm our present results. The locomotor activity of adult Wistar rats in "open field" within 20 min starting 25 min after intraperitoneal administration of JMV2959 (1, 3 and 6 mg/kg i.p.) did not significantly differ from the control group which was treated with saline (N = 9).

Locomotor activity in the "open field" (Jerabek et al 2017	DISTANCE (20 min interval)	VELOCITY (20 min interval)
Saline + saline	873.33 ± 142.34	0.73 ± 0.12
JMV2959 1 mg/kg + saline	950.78 ± 102.44	0.79 ± 0.09
JMV2959 3 mg/kg + saline	722.32 ± 80.05	0.60 ± 0.07
JMV2959 6 mg/kg + saline	720.00 ± 68.44	0.60 ± 0.06
One-way ANOVA (N = 9)	F3,32 = 0.954, P = 0.426 (n.s.)	F3,32 = 0.954, P = 0.426 (n.s.)

Table S3. Locomotor activity in "open field" within 20 min starting 25 min after intraperitoneal administration of JMV2959 (1, 3 and 6 mg/kg i.p.). Selected data from our previous study (Jerabek et al. 2017).

The cannabinoid mechanisms of effect and the endocannabinoid system

Cannabinoids including the main cannabis constituent tetrahydrocannabinol/THC and synthetic cannabinoids e.g. WIN55,212-2, most likely mediate their pleasurable, anxiolytic and rewarding/reinforcing effects through the CB1Rs located within the central brain reward circuits, particularly the VTA and the NAC (Herkenham 1991; Matsuda et al. 1990; Parsons and Hurd 2015). Cannabinoids activate these reward pathways in a manner that is consistent with other drugs of abuse, including the trigger dopamine release in the nucleus accumbens shell (NACSh)(Bloomfield et al. 2016; Gardner 2005; Parsons and Hurd 2015; Tanda et al. 1997; Vlachou and Panagis 2014; Volkow et al. 2017; Zehra et al. 2018), however, particular mechanisms through which this occurs, seem to be more specific; it would appear that THC (and probably other exogenous cannabinoids) may have pathway-dependent effects in this brain reward circuit (further research is needed)(Covey et al. 2015; Hoffman and Lupica 2013).

The CB1R-dependent effects emerge only during periods of heightened cellular activity. The GABA inhibition is sufficient to induce the dopamine neuron bursting (Lobb et al. 2010), so, CB1 receptor activation on the VTA GABA terminals facilitates these actions and CB1 agonists generate dopamine cell firing and phasic accumbens dopamine release hence reward seeking (Cheer et al. 2003; Cheer et al. 2004); particularly, involvement of GABA VTA inputs from the rostromedial tegmental nucleus (RMTg) were suggested (Lecca et al. 2012; Melis and Pistis 2012). Bursting VTA dopamine neurons synthesize and release endocannabinoids, which through retrograde CB1 activations depolarisation-induced suppression of inhibition (DSI, e.g. reduced GABA release) enhances/drives dopamine release. The VTA 2-AG seems to play the main role in the dopamine activation, on the contrary, anadamide/AEA impeded the CB1R binding of 2-AG and antagonises its effects in the VTA (Covey et al. 2015; Volkow et al. 2017). The role of CB2R is still unclear.

It was described in rats that a single exposure to THC can cause a selective remodelling of a population of glutamatergic synaptic inputs to dopamine neurons in the VTA. Specifically, THC rapidly increased the expression of AMPA receptors with the GluA1 subunit selectively at subcortical pedunculopontine nucleus (PNN) glutamatergic inputs to VTA dopamine neurons. This is thought to result in long-term potentiation (LTP) of these synapses and increase the subcortical drive of VTA DA neurons by the PPN and may play a role in long-term modification in mesolimbocortical dopaminergic output. In the contrary, cocaine-induced glutamatergic synapses remodelling in the VTA was not selective, it was observed also on non-

PNN inputs (similarly to other abused drugs) (Good and Lupica 2010; Hoffman and Lupica 2013).

The cannabinoid-induced (phasic) dopamine release within the NACSh activates direct GABAergic medium spiny neurons (dMSNs) through excitatory D1Rs and inhibits the indirect MSNs/iMSNs through inhibitory D2Rs. The D1Rs have low affinity for dopamine, thus they are preferentially activated by large surges/phasic release of dopamine and result in the activation of the direct pathway (dMSNs), which project to the midbrain regions including the VTA that in extend increases approach/appetitive behaviour. The D2Rs have a high affinity for dopamine, thus, basal extracellular dopamine concentrations occupy the D2Rs and inhibit the iMSNs output and supress indirect pathway that projects to the ventral pallidum, thus inhibiting the avoidance behaviour. If there is a dopamine concentration decrease (following exposure to aversive stimulus), then it would relieve the D2R-mediated suppression of iMSNs and promote avoidance behaviour. Therefore, the reward-related phasic dopamine release increases the approach behaviour and reduces the avoidance behaviour (Covey et al. 2015; Parsons and Hurd 2015).

The NACSh MSN activity is substantially modulated by glutamatergic inputs from the prefrontal cortex (PFC), basolateral amygdala and ventral hippocampus that express CB1Rs. Activated accumbens MSN cells and probably fast spiking interneurons (FSI) (Schall et al. 2020) produce and release endocannabinoids (2-AG, AEA), which both modulate presynaptic axons within the NACSh. The CB1-mediated suppression of (glutamate) excitatory signalling is preferentially active at iMSN synapses possibly resulting from D2R-mediated endocannabinoid production (2-AG, AEA) from the iMSN cell bodies. Thus, increased endocannabinoid formation preferentially reduces excitatory inputs to iMSNs (in comparison to dMSNs) which results in decreasing avoidance.

Single acute administration of THC in mice caused a CB1-dependent impairment of the long term depression (LTD) of synaptic transmission at excitatory/glutamatergic synapses on the MSNs in the NACSh (Mato et al. 2004), resulting in diminished constraint of glutamatergic release and increased excitation of NACSh cells.

Thus, the THC/cannabinoid single exposure impairs the endocannabinoid-mediated plasticity in the VTA and the NAC, induces an overall enhancement of mesolimbic signalling, and increase in the sensitivity of dopamine neurons to subcortical excitatory drive in specific set of synapses, which may alter motivational processes mediated by the NAC (Covey et al. 2015; Hoffman and Lupica 2013; Panlilio et al. 2015; Parsons and Hurd 2015; Volkow et al. 2017). (The repeated/chronic cannabinoid effects are not discussed in this article).

Figure S1. Administration of JMV2959 1 mg/kg i.p. alone also did not induce significant changes in the accumbens dopamine and metabolites (3-MT, DOPAC, HVA) as it is illustrated in the Figure S1.

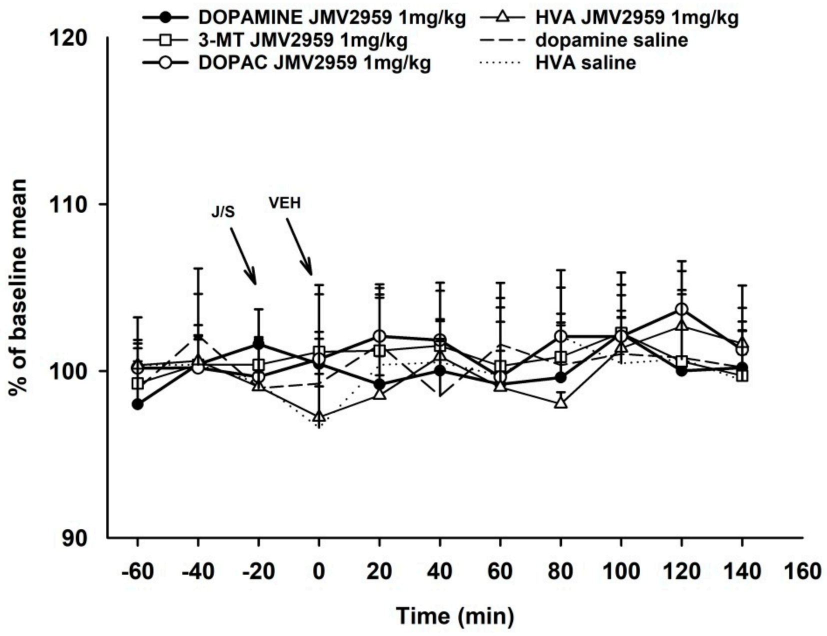

Figure S1: Effects of JMV2959 1 mg/kg alone (N = 4) on the accumbens extracellular concentrations of dopamine (full circle), 3-MT (open square), DOPAC (open circle), HVA (open triangle) in comparison to saline (N = 7) effects on accumbens dopamine (dashed line) and HVA (dotted line). No significant differences were found among the groups.

Bloomfield MA, Ashok AH, Volkow ND, Howes OD (2016) The effects of Delta(9)-tetrahydrocannabinol on the dopamine system. Nature 539: 369-377.

Covey DP, Wenzel JM, Cheer JF (2015) Cannabinoid modulation of drug reward and the implications of marijuana legalization. Brain research 1628: 233-43.

Gardner EL (2005) Endocannabinoid signaling system and brain reward: emphasis on dopamine. Pharmacol Biochem Behav 81: 263-84.

Good CH, Lupica CR (2010) Afferent-specific AMPA receptor subunit composition and regulation of synaptic plasticity in midbrain dopamine neurons by abused drugs. J Neurosci 30: 7900-9.

Herkenham M (1991) Characterization and localization of cannabinoid receptors in brain: an in vitro technique using slide-mounted tissue sections. NIDA Res Monogr 112: 129-45.

Hoffman AF, Lupica CR (2013) Synaptic targets of Delta9-tetrahydrocannabinol in the central nervous system. Cold Spring Harbor perspectives in medicine 3.

Cheer JF, Kendall DA, Mason R, Marsden CA (2003) Differential cannabinoid-induced electrophysiological effects in rat ventral tegmentum. Neuropharmacology 44: 633-41.

Cheer JF, Wassum KM, Heien ML, Phillips PE, Wightman RM (2004) Cannabinoids enhance subsecond dopamine release in the nucleus accumbens of awake rats. J Neurosci 24: 4393-400.

Jerabek P, Havlickova T, Puskina N, Charalambous C, Lapka M, Kacer P, Sustkova-Fiserova M (2017) Ghrelin receptor antagonism of morphine-induced conditioned place preference and behavioral and accumbens dopaminergic sensitization in rats. Neurochemistry international 110: 101-113.

Lecca S, Melis M, Luchicchi A, Muntoni AL, Pistis M (2012) Inhibitory inputs from rostromedial tegmental neurons regulate spontaneous activity of midbrain dopamine cells and their responses to drugs of abuse. Neuropsychopharmacology 37: 1164-76.

Lobb CJ, Wilson CJ, Paladini CA (2010) A dynamic role for GABA receptors on the firing pattern of midbrain dopaminergic neurons. J Neurophysiol 104: 403-13.

Mato S, Chevaleyre V, Robbe D, Pazos A, Castillo PE, Manzoni OJ (2004) A single in-vivo exposure to delta 9THC blocks endocannabinoid-mediated synaptic plasticity. Nat Neurosci 7: 585-6.

Matsuda LA, Lolait SJ, Brownstein MJ, Young AC, Bonner TI (1990) Structure of a cannabinoid receptor and functional expression of the cloned cDNA. Nature 346: 561-4.

Melis M, Pistis M (2012) Hub and switches: endocannabinoid signalling in midbrain dopamine neurons. Philos Trans R Soc Lond B Biol Sci 367: 3276-85.

Panlilio LV, Goldberg SR, Justinova Z (2015) Cannabinoid abuse and addiction: Clinical and preclinical findings. Clin Pharmacol Ther 97: 616-27.

Parsons LH, Hurd YL (2015) Endocannabinoid signalling in reward and addiction. Nat Rev Neurosci 16: 579-94.

Schall TA, Wright WJ, Dong Y (2020) Nucleus accumbens fast-spiking interneurons in motivational and addictive behaviors. Molecular psychiatry.

Tanda G, Pontieri FE, Di Chiara G (1997) Cannabinoid and heroin activation of mesolimbic dopamine transmission by a common mu1 opioid receptor mechanism. Science 276: 2048-50.

Vlachou S, Panagis G (2014) Regulation of brain reward by the endocannabinoid system: a critical review of behavioral studies in animals. Curr Pharm Des 20: 2072-88.

Volkow ND, Hampson AJ, Baler RD (2017) Don't Worry, Be Happy: Endocannabinoids and Cannabis at the Intersection of Stress and Reward. Annu Rev Pharmacol Toxicol 57: 285-308.

Zehra A, Burns J, Liu CK, Manza P, Wiers CE, Volkow ND, Wang GJ (2018) Cannabis Addiction and the Brain: a Review. J Neuroimmune Pharmacol 13: 438-452.
